# Overexpression of stathmin plays a pivotal role in the metastasis of esophageal squamous cell carcinoma

**DOI:** 10.18632/oncotarget.18687

**Published:** 2017-06-27

**Authors:** Gaijing Han, Zongyong Wu, Nan Zhao, Lanping Zhou, Fang Liu, Fangfei Niu, Yang Xu, Xiaohang Zhao

**Affiliations:** ^1^ State Key Laboratory of Molecular Oncology, National Cancer Center/Cancer Hospital, Chinese Academy of Medical Sciences and Peking Union Medical College, Beijing, China; ^2^ Clinical Laboratory, National Cancer Center/Cancer Hospital, Chinese Academy of Medical Sciences and Peking Union Medical College, Beijing, China

**Keywords:** stathmin, integrinα5β1, FAK, ERK, ESCC

## Abstract

**Purpose:**

Esophageal squamous cell carcinoma (ESCC) is a serious malignant tumor that affects human health. We analyzed the correlation between serum stathmin level and ESCC and elucidated the molecular mechanisms of stathmin's promotion of ESCC cell invasion and metastasis.

**Methods:**

Stathmin level in ESCC and healthy control serum were detected by enzyme-linked immunosorbent assay (ELISA), and the clinical parameters were analyzed. We established ESCC cells with stathmin overexpression or knockdown and then evaluated the effects of stathmin on invasion and metastasis in ESCC. Differentially expressed genes were analyzed by Human Transcriptome Array and confirmed by RT-PCR. The expression levels of the integrin family, focal adhesion kinase (FAK) and extracellular signal-regulated kinase (ERK) were detected by immunoblotting.

**Results:**

Serum levels of stathmin were significantly higher in ESCC than in control serum and associated with lymph node metastasis, tumor stage and size. Furthermore, we found that stathmin promoted migration and invasion of ESCC cells *in vitro* and *in vivo*. In addition, we confirmed that the activation of the integrinα5β1/FAK/ERK pathway is increased in stathmin-overexpression cells and accelerates cell motility by enhancing cell adhesion ability.

**Conclusion:**

Stathmin may predict a potential metastasis biomarker for ESCC.

## INTRODUCTION

Esophageal cancer (EC) is the eighth most common malignant cancer and the sixth most common cause of cancer deaths worldwide; it seriously affects human health [1]. Esophageal squamous cell carcinoma (ESCC) accounts for approximately 90% of EC cases in developing countries. The prevalence of ESCC varies geographically and is highest in China, Southeast Africa and Japan [1, 2]. The clinical features of ESCC include a lack of obvious early symptoms, a lack of serum detection markers of sufficient sensitivity and specificity, and poor prognosis. Current treatment for ESCC includes surgical resection, radiotherapy and chemotherapy [3]. Metastasis is primarily responsible for ESCC mortality, yet the molecular mechanism of metastatic dissemination remains unclear [4]. Clinical studies have found that ESCC spreads through the lymph and blood; the most common metastatic site is the lung, followed by the liver, bone and brain. Most patients have local or distant lymph node metastases at surgery [5, 6].

The microtubule is involved in many biological processes, such as cell cycle progression, cell proliferation, intracellular signal transduction, the transport of substances and cell movement [7]. Stathmin is a microtubule-destabilizing protein and is also known as oncoprotein18 (Op18); it binds to α, β-tubulin heterodimers and regulates microtubule dynamics [8, 9]. In 1982, Schubart found stathmin in hamster insulinoma cells. In 1988, stathmin was found to be overexpressed in acute leukemia cells, and this discovery was the first identified association of stathmin with human malignancies [10, 11]. There is increasing evidence that stathmin is related to a variety of tumors, such as endometrial, breast, melanoma, gastric, ovarian and bladder cancers. Stathmin is highly expressed in cancerous tissues, and its level is associated with tumor drug resistance, metastasis, recurrence, and prognosis [12–18]. Therefore, stathmin may be a therapeutic target for a variety of tumors.

Previously, our laboratory used matrix-assisted laser desorption/ionization time of flight mass spectrometry (MALDI-TOF-MS) to analyze the differential proteomic expression profiles of 8 pairs of ESCC and adjacent normal tissues. The results showed that the expression of stathmin was higher in ESCC tissues than in adjacent normal tissues. We then analyzed stathmin expression in 143 ESCC tissues using immunohistochemistry. The results indicated that stathmin expression was relatively high in ESCC tissues and negatively correlated with the degree of ESCC differentiation [19]. Wang et al. also found stathmin overexpression in ESCC, which was associated with lymph node metastasis, TNM stage and other clinical parameters [20]. Akhtar et al. demonstrated that the 5-year survival was significantly lower in ESCC patients with high stathmin than in those with low stathmin, which indicated that stathmin could be used as a marker for ESCC prognosis [21, 22]. However, studies on the mechanisms of overexpression and the serum levels in ESCC are limited. We investigated whether stathmin overexpression is associated with metastasis and whether stathmin serum level represents a potential tumor marker for ESCC.

In this study, our main goals are to evaluate the serum level of stathmin and explore the previously uncharacterized role of stathmin overexpression in promoting the migration and invasion of esophageal cancer cells. In addition, we considered whether stathmin could be a potential metastasis marker for ESCC diagnosis.

## RESULTS

### Clinical significance of serum stathmin in ESCC

**Figure 1:**
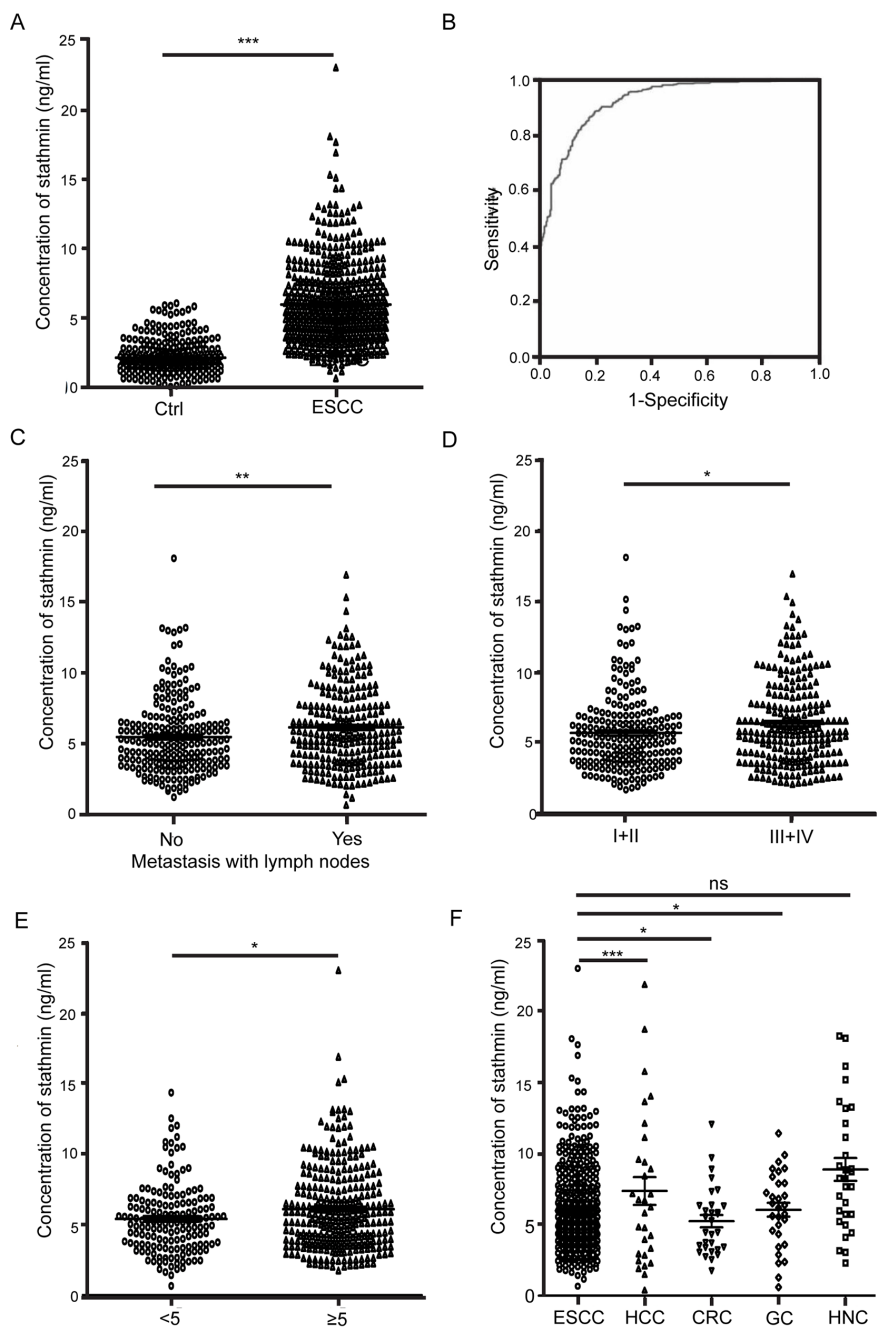
Clinical significance of serum stathmin in ESCC **(A)** The concentration of stathmin in ESCC patients was significantly higher than that in healthy controls (*P*<0.0001). **(B)** ROC curve analysis of the sensitivity and specificity of serum stathmin in ESCC and healthy controls (*P*<0.005). **(C)** Stathmin levels in ESCC lymph node metastasis were significantly higher than those in non-metastatic (*P*<0.05). **(D)** Stathmin levels in ESCC stage III+IV were higher than those in stage I+II (*P*<0.005). **(E)** Comparison of stathmin levels among different cancers, include ESCC, hepatocellular carcinoma (HCC), colorectal cancer (CRC), gastric cancer (GC) and head and neck cancer (HNC).

ELISA analysis showed that the concentration of stathmin in ESCC patients was significantly elevated (n=535, mean 5.98 ng/ml, standard deviation (SD) ±2.89 ng/ml) compared with the concentration in healthy controls (n=288, mean 2.16 ng/ml, SD±1.19 ng/ml, *P* < 0.001) (Figure 1A). Receiver operating characteristic (ROC) curves were used to establish the sensitivity-specificity relationship for stathmin (Figure 1B). The area under the curve (AUC) was 0.924, and the cut-off level determined by the Youden index was 3.014 ng/ml. The sensitivity of stathmin in detecting ESCC was 88.6% at a specificity of 80.6%. The correlation analysis of clinic pathological data with stathmin (Table 1) using cross tabulation in 535 patients showed a significant positive association between stathmin level and tumor size (>5 cm vs. <5 cm) (6.10±3.00 ng/ml vs. 5.41±2.43 ng/ml, *P*<0.05). Significantly higher levels were observed in patients with lymph node metastasis than in those without (6.14±2.85 ng/ml vs. 5.67±2.94 ng/ml, *P*<0.005) (Figure 1C). ELISA also showed that stathmin expression escalated with clinical stage from I+II to III+IV (6.40±2.91 ng/ml vs. 5.72±2.75 ng/ml, *P*<0.05) (Figure 1D). Additionally, serum levels of stathmin were measured in other patients include head and neck cancer, colorectal cancer, gastric cancer and hepatocellular carcinoma, the results showed that the stathmin concentration was higest in head and neck cancer (8.88±4.34 ng/ml) and lowest in colorectal cancers (5.24±2.34 ng/ml) (Figure 1E). In conclusion, these results suggest that stathmin may act as a biomarker for the clinical detection of ESCC and can be used in the diagnosis of lymph node metastasis.

**Table 1:** Correlation between serum stathmin levels and clinicalpathological features

Parameter	Group	N	Levels serum stathmin (ng/ml)	*P*
			Mean ±SD	
ESCC patients		535	5.98±2.89	<0.001
Healthy control		288	2.16±1.19	
Lymph nodes metastases				
	Yes	221	5.48±2.61	<0.005
	No	248	6.14±2.85	
Tumor stage				
	I+ II	209	5.72±.72I	<0.05
	III+IV	233	6.40±2.91	
Tumor size				
	<5cm	158	5.41±2.43	<0.05
	≥5cm	302	6.10±3.00	
Pathological grade				
	poorly	73	6.25±2.88	0.337
	Moderately	267	5.91±2.91	
	well	118	5.89±2.95	
Gender				
	Male	438	6.18±2.91	<0.001
	Female	97	5.08±2.61	
Ages				
	≥50	477	5.88±2.86	<0.05
	<50	58	6.78±3.0	

### Stathmin overexpression promotes ESCC cell metastasis

**Figure 2:**
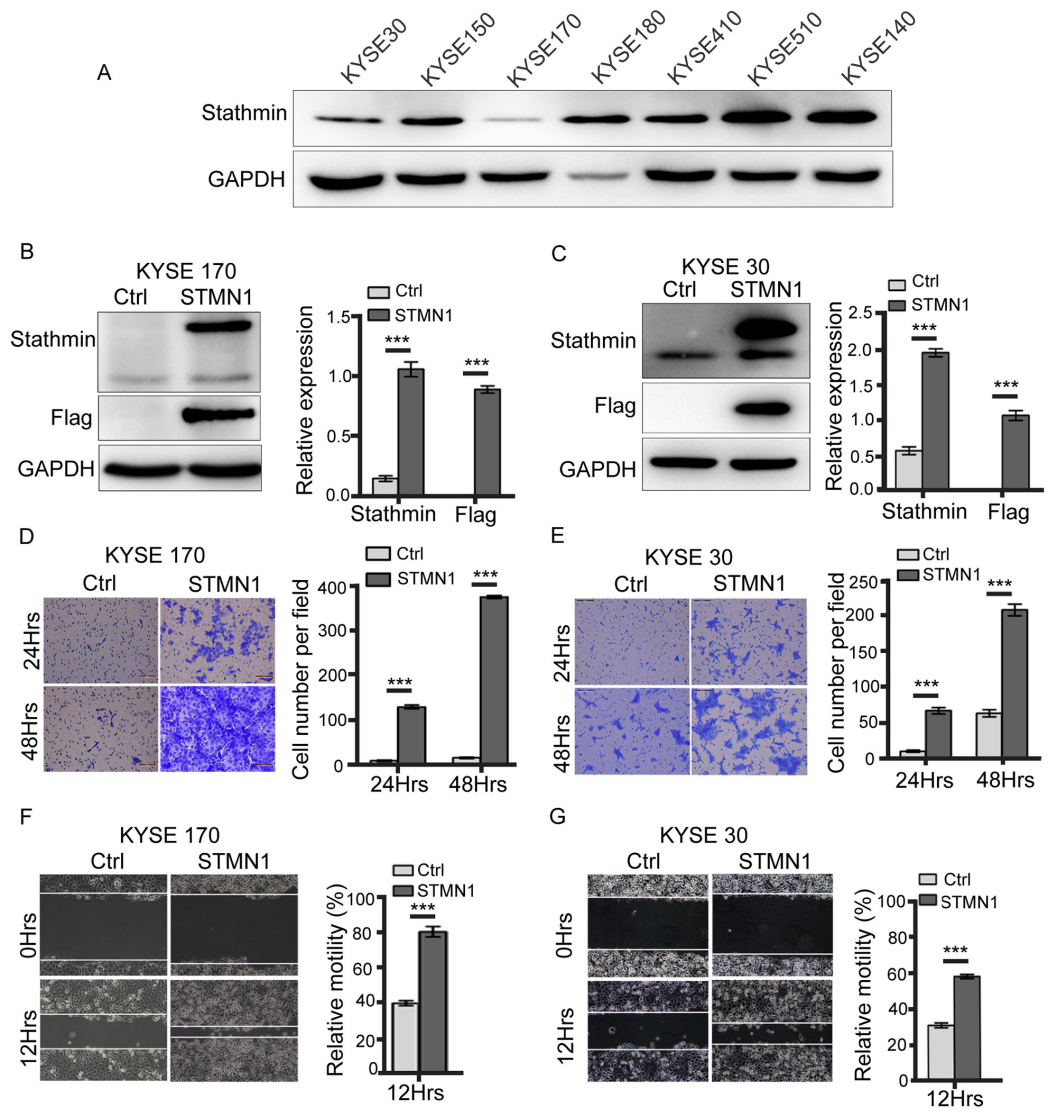
Stathmin promoted ESCC cell invasion and migration **(A)** Stathmin expression in seven ESCC cell lines was examined by western blotting. GAPDH was used as a loading control. **(B, C)** Immunoblotting was used to analyze the stathmin protein level in KYSE 170 and KYSE 30 cells. Control (Ctrl) represents KYSE 170 or KYSE 30 cells transfected with the control plasmid; STMN1 represents KYSE 170 or KYSE 30 cells transfected with STMN1-plasmid. **(D, E)** The transwell invasion system demonstrated an enhanced invasion capacity of the KYSE 170 and KYSE 30 stathmin-overexpression groups compared with controls. Images of invading cells were captured by phase contrast microscopy at 200× magnification. The y-axis represents the number of invading cells. **(F, G)** A wound-healing assay was performed to investigate the migratory potential of KYSE 170 and KSYE 30 cells after stathmin levels changed. In the quantitative migration assay results, the y-axis represents the migration rate relative to that of control cells. Stathmin overexpression significantly promoted the migration ability of both cell lines. All assays were replicated, and results are presented as the mean±SD (*, *P*<0.05).

To confirm the role of stathmin overexpression, seven human esophageal cancer cells were tested by western blot. As shown in Figure 2A, among the cell lines, the KYSE 30 and KYSE 170 ESCC cells showed the lowest stathmin expression. Therefore, the KYSE 30 and KYSE 170 cells were used for the overexpression experiments and KYSE 510 cell were knocked down. After transfection of KYSE 30 and KYSE 170 cells with the STMN1 plasmid (KYSE 30-STMN1/KYSE 30-Ctrl, KYSE 170-STMN1/KYSE 170-Ctrl), we found overexpression of stathmin compared with the expression level of the control group (Figure 2B and 2C). KYSE 510 cells were used for stathmin knocked down experiments. After transfection with two different STMN1 specific siRNAs (siRNA-1 and siRNA-2), a reduction in stathmin protein expression was observed relative to negative control (NC) siRNA-treated cells (Supplementary Figure 1A).

To determine whether stathmin level might affect cellular invasion, transwell assays were performed. As shown in Figure 2D and 2E, stathmin overexpression was associated with significantly increased cellular invasion compared with the level in the control group. In contrast, STMN1-knockdown by siRNA in KYSE510 cells effectively reduced the populations of transwell invasive cells compared with the populations in the control group (Supplementary Figure 1B and 1C). Relative motility was tested by wound-healing assay, as shown in Figure 2F and 2G. Stathmin overexpression promoted cell migration in KYSE170 and KYSE30 cells, which overexpressed stathmin (Figure 2B and 2C). Furthermore, in knockdown STMN1 experiments, the relative motility of cells treated with STMN1-siRNA was significantly decreased compared with that of the NC group (Supplementary Figure 1C). To determine the ability of a cell to proliferate indefinitely after an alteration in stathmin level, we performed a colony-formation assay with KYSE170 cells. The colony-formation capacity was enhanced in KYSE 170 cells compared with that in the control group (Supplementary Figure 2A). To confirm that the observed increase in migration was not in fact an increase in cell proliferation, mitomycin C was used to inhibit cell proliferation during the cell wound-healing assay. We found that the relative motility of the STMN1 group was significantly increased compared with the control group (Supplementary Figure 2B). Taken together, our results indicated that stathmin overexpression enhanced the invasion, migration and proliferation of ESCC cells.

### Gene expression changes in ESCC cells with stathmin overexpression

**Figure 3:**
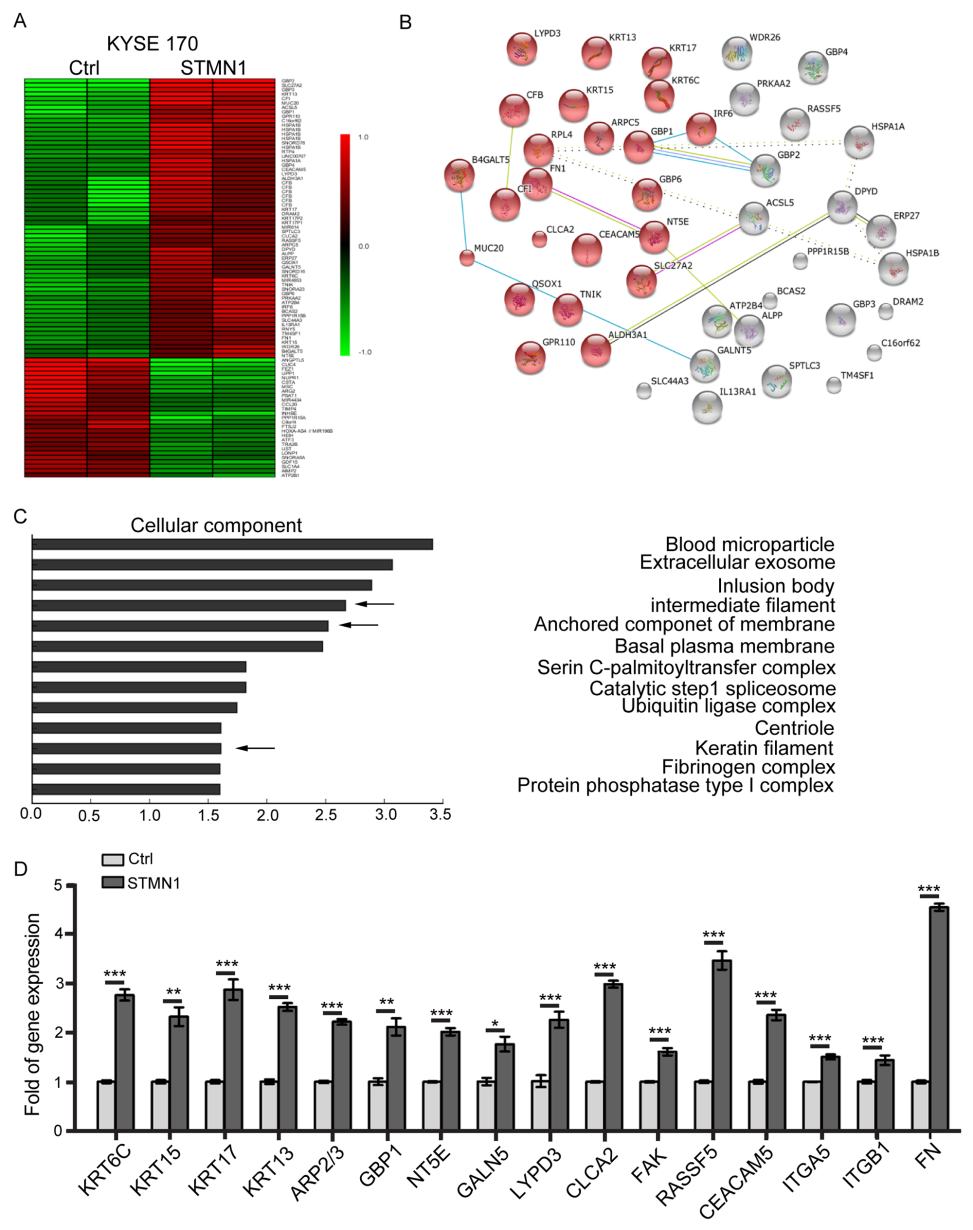
Gene expression changes in stathmin-overexpressing ESCC cells **(A)** Heatmap representation of 90 genes showing significant differential expression between the STMN1 group and the control group. A color scale for the normalized expression data is shown on the right side of the microarray heatmap (green represents downregulated genes, whereas red represents upregulated genes). **(B)** The gene changes were functionally analyzed using the online tool STRING. **(C)** The upregulated genes were functionally classified based on their cellular components using the DAVID functional annotation clustering tool. **(D)** The mRNA levels of candidate genes were determined using real-time PCR. The data represent the mean±SD of relative mRNA levels versus control cells. (*, *P*<0.05; **, *P*<0.01; ***, *P*<0.001).

To characterize the molecular pathways affected by stathmin in ESCC cells, we used the Human Transcriptome Array (HTA2.0) to perform microarray-based global gene expression profiling of KYSE 170 cells with exogenous increased stathmin expression. Global gene expression profiling of KYSE 170-Ctrl and KYSE 170-STMN1 cells was conducted using a microarray platform. Significantly differential genes expression were found, including 63 upregulated and 27 downregulated genes (Figure 3A). The results of the STRING enrichment analysis are shown in Figure 3B. Sixty-three upregulated genes (indicated by red on the heat map) were found to be functionally enriched in gene ontology categories extracellular region and cellular component. Using the DAVID database, we found that stathmin might increase a wide range of cellular adhesion molecules, which would be consistent with the higher malignancy of cancer cells with stathmin overexpression (Figure 3C). To validate differentially expressed genes in KYSE170 cells, several genes associated with cell adhesion and cytoskeleton were verified by real-time PCR, including KRT6C, KRT15, KRT17, KRT13, ARP2/3, GBP1, NT5E, GALN5, FN, LYPD3, CLCA2, FAK, RASSF5 and CEACAM5. The mRNA levels of FN, LYPD3, CLCA2, FAK, RASSF5 and CEACAM5 were highly upregulated in the STMN1 group (Figure 3D).

**Figure 4:**
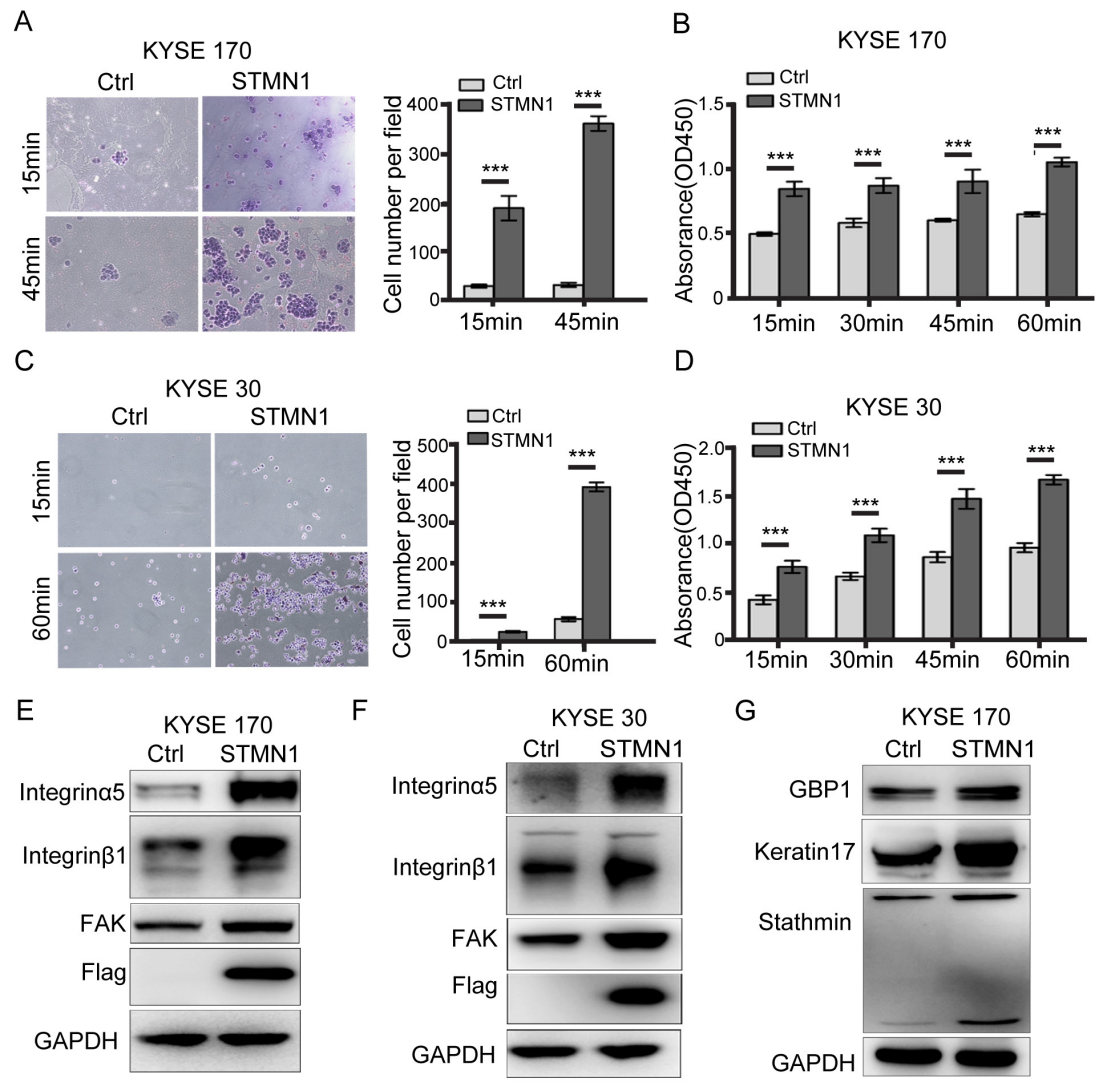
Stathmin increased ESCC cell adhesion to FN by promoting integrinα5β1/FAK expression **(A, C)** Adhesion experiments showed that the level of cell adhesion to the FN-coated slides was significantly increased in the STMN1 group compared with that in the control group. **(B, D)** Adhesion experiments using FN-coated 96-well plates showed that the absorbance value of the STMN1 group was significantly higher than that of the control group. **(E, F)** The protein levels of integrinα5β1 and FAK were much higher in the STMN1 group than in the control group. **(G)** Immunoblotting identified differential keratin17 and GBP1 protein expression between theSTMN1 group and Ctrl group (***, *P*<0.001).

KRT17 and GBP1 genes were also tested for protein expression by western blot in the STMN1 and control group cells. The protein levels of the KRT17 and GBP1 genes were increased in the cells with stathmin overexpression (Figure 4G). These findings suggested that stathmin overexpression might affect cell adhesion and intermediate filaments by increasing the number of cellular adhesion molecules. Cytokeratin 17 (KRT17) is a basal cell keratin involved in the development and metastasis in ESCC of the esophagus [23, 24]. We investigated the stability of Keratin 17 via immune fluorescence staining in stathmin-overexpressing KYSE170 cells. After treatment with 2% FBS medium for 6 h, the intermediate filaments of the cells with stathmin overexpression maintained their shape, bearing tension and retaining rigidity; however, most of those of the control cells shrank and disintegrated (Supplementary Figure 3A and 3B). In addition, knockdown of KRT17 decreased cell mobility in KYSE 170-STMN1 cells (Supplementary Figure 4A-4C). These results suggest that stathmin overexpression enhances the stability of intermediate filaments in the starvation.

### Stathmin overexpression enhances cell adhesion and the integrinα5β1/FAK signaling pathway

The transcriptome data showed that fibronectin (FN) and adhesion-related molecules were significantly increased in the STMN1 group. To evaluate cell adhesion ability, FN adhesion experiments were performed in cells with stathmin overexpression. As shown in Figure 4A and 4C, cell attachment was time dependent. The levels of cell adhesion and absorbance were significantly increased in the cells with stathmin overexpression (Figure 4B and 4D). In contrast, knockdown of STMN1 led to decreased cell adhesion in KYSE 510 cells (Supplementary Figure 5A). These data indicated that stathmin overexpression facilitated cell adhesion in ESCC cells. Integrinα5β1 is the primary cell surface receptor for FN [25, 26], and integrin and focal adhesion kinase (FAK) are involved in cell adhesion and migration [27–29]. To address the contribution of the enhanced cell adhesion, we analyzed protein expression of the integrin family and FAK by western blotting in the STMN1 and control groups. As shown in Figure 4E and 4F, the total amounts of integrinα5β1 and FAK were significantly increased in stathmin-overexpressing KYSE170 and KYSE 30 cells. However, the integrinβ3, 4, and 5 and vinculin and p-vinculin levels did not significantly differ between the STMN1 group and the control group (Supplementary Figure 6A). Following knockdown of stathmin by siRNA, the protein levels of integrinα5β1 and FAK were greatly reduced in the KYSE 510 and KYSE 170-STMN1 cells (Figure 8E and 8F). These results demonstrate that stathmin increases the protein expression of integrinα5β1 and FAK.

**Figure 5:**
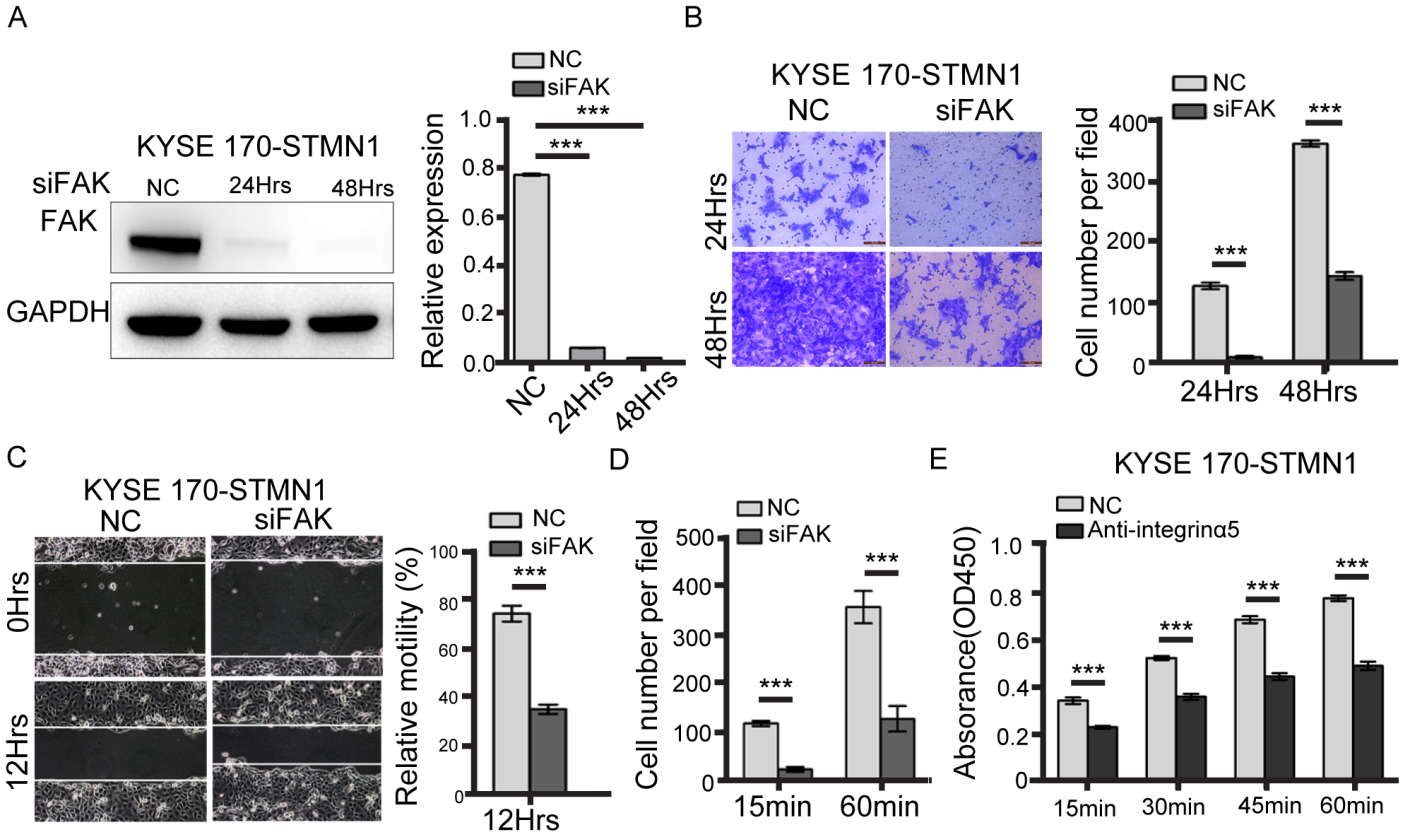
FAK knockdown inhibited metastasis of stathmin-overexpressing cells **(A)** Western blotting showed that FAK-specific siRNA decreased FAK expression. **(B)** The transwell assay revealed that the invasion number of the FAK-knockdown group was significantly decreased compared with that of the NC group. **(C)** The wound-healing assay was performed to detect the migratory potential of FAK-knockdown KYSE 170-STMN1 cells and showed that the motility of the FAK-knockdown group was decreased compared with that of the NC group. **(D)** The cell adhesion assay to investigate cell adhesion ability showed that the level of cell adhesion to FN was lower in the FAK-knockdown group than in the NC group. **(E)** Cells were incubated with 10 ug/ml antibody against integrinα5 45min before plating on FN. Histograms represent the absorbance (OD450) (***, *P*<0.001).

**Figure 6:**
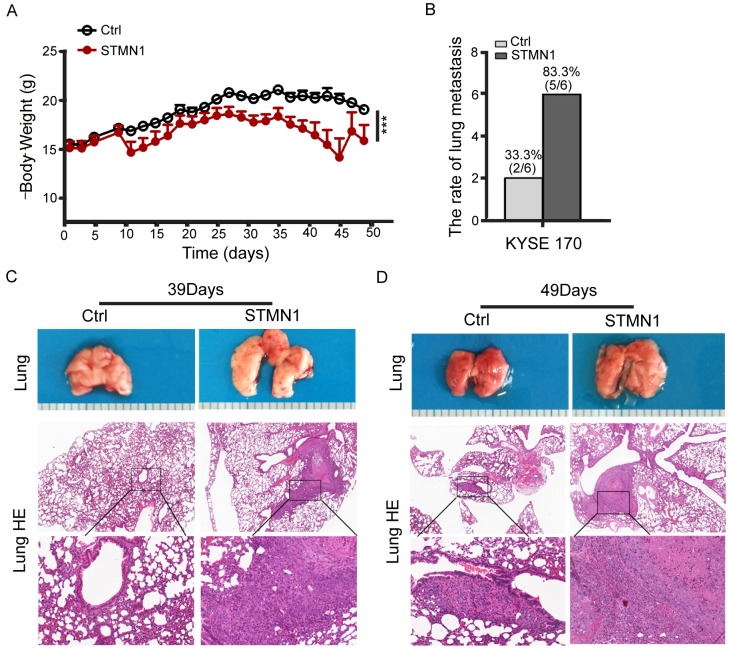
Stathmin overexpression increased ESCC cell lung metastasis *in vivo* **(A)** ESCC cells were implanted into mouse tail veins, and body weight was measured every two days. Body weight was significantly lower in mice injected with STMN1 cells than in the control group (*P*<0.001). **(B)** Statistical results of lung metastasis. **(C)** After 39 days, the STMN1 cell-injected mice had developed metastatic nodules, whereas the control group had not. **(D)** After 49 days, both groups of ESCC cell-injected mice developed metastatic nodules, although the lung metastasis area was larger in the STMN1-injected mice.

**Figure 7:**
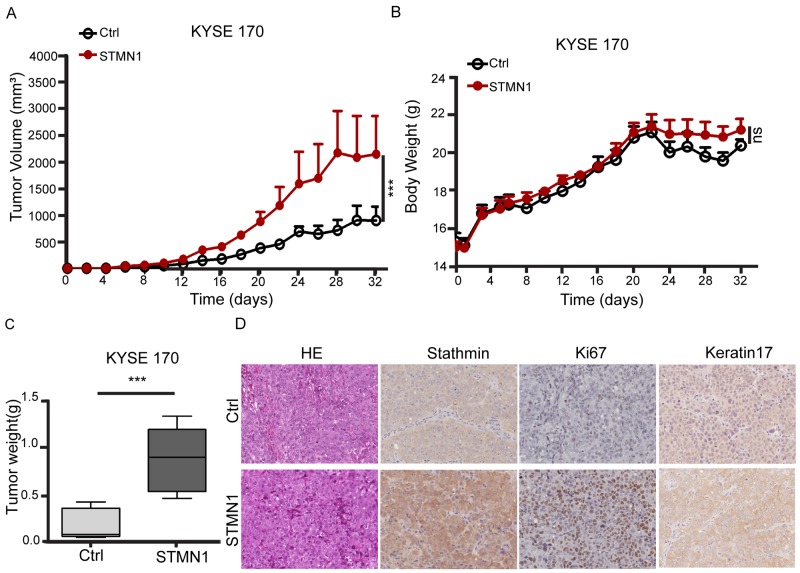
Stathmin overexpression increased xenografted tumor growth **(A, C)** Control cells and STMN1 cells were orthotopically inoculated into nude mice. Tumor volume and tumor weight significantly increased in mice injected with STMN1 group compared with the control group. **(B)** Body weight was measured every two days after injection, and no significant difference was observed between the two groups. **(D)** IHC staining showed strong staining in the tissue sections for stathmin, Ki67 and keratin 17 in the STMN1 group.

**Figure 8:**
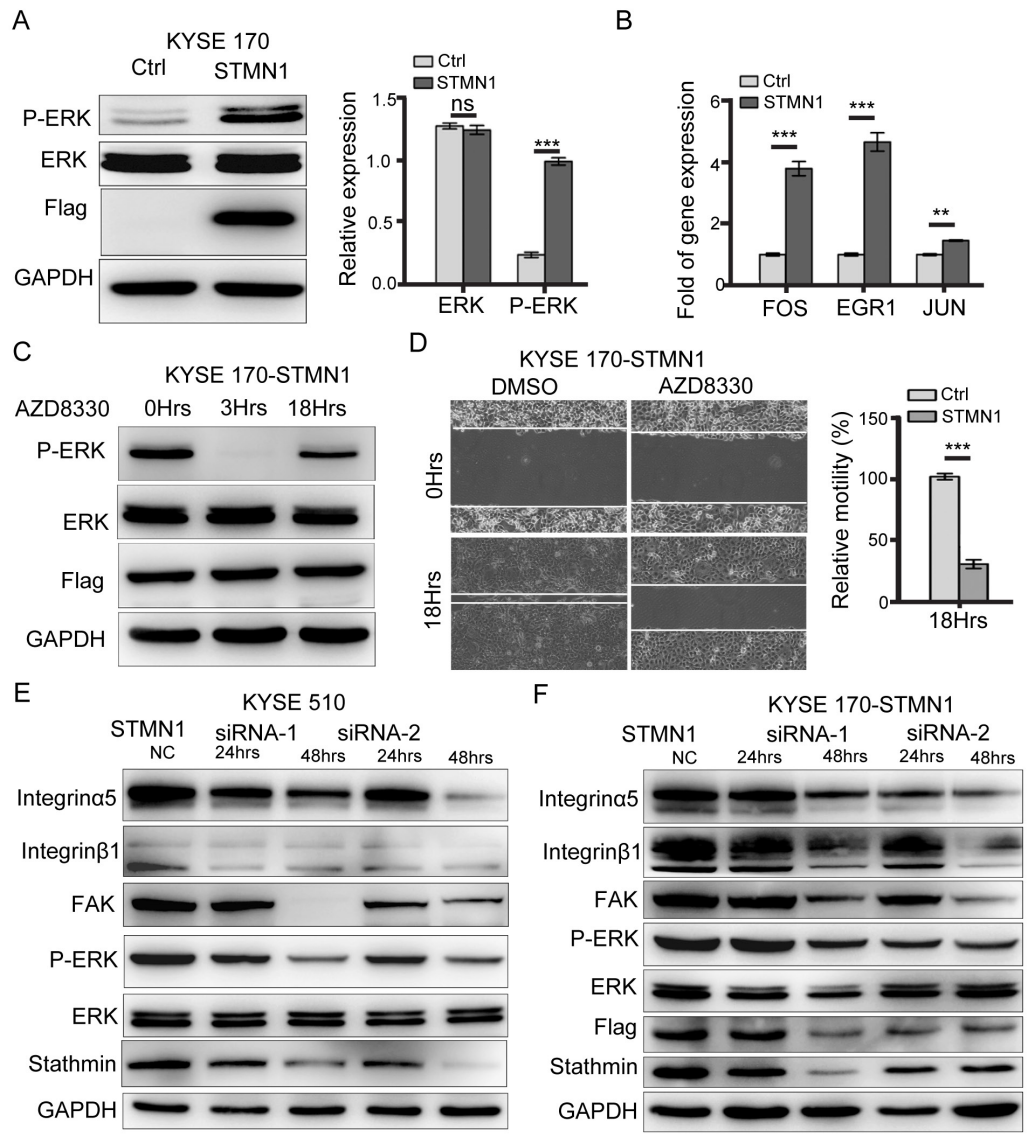
Stathmin regulated ERK activation **(A)** Western blot analysis showed that the protein level of P-ERK in the STMN1 group was significantly higher than that of the control group. **(B)** RT-PCR analysis of the mRNA levels of ERK downstream transcription factors such as FOS, EGR1, and JUN revealed significantly higher levels in the STMN1group than in the control group. **(C)** KYSE 170-STMN1 cells were treated with ERK inhibitors AZD8330; western blot analysis showed that ERK phosphorylation was inhibited. **(D)** The effects of ERK phosphorylation on the migration ability of KYSE 170-STMN1 cells were analyzed by wound-healing assay. The results showed that inhibition of ERK phosphorylation in KYSE 170-STMN1 cells markedly reduced cell motility. **(E)** Stathmin expression in KYSE 510 and **(F)** KYSE 170-STMN1 cells was knocked down by two different STMN1-specific siRNAs (siRNA-1 and siRNA-2), and the activation of the integrinα5β1/FAK/ERK pathway was measured by immunoblotting (ns, *P*>0.05; *, *P*<0.05; ***, *P*<0.001).

To further evaluate the potential contribution of the integrinα5β1/FAK pathway in stathmin-overexpressing cells, we performed wound-healing and transwell assays after FAK knockdown by siRNA in KYSE 170-STMN1 cells. The western blotting results, shown in Figure 5A, revealed that FAK was strongly knocked down in KYSE 170-STMN1 cells. The motility and population of invasion cells were significantly reduced compared with those of the NC group (Figure 5B and 5C). In addition, cell adhesion was mitigated at a different time point from that of the NC group (Figure 5D). In KYSE 170-STMN1 cells treated with integrinα5 antibody, attenuated cell attachment was observed compared with cell attachment in the control group, as shown in Figure 5E. These data indicate that stathmin overexpression may increase cell adhesion and migration through the integrinα5β1/FAK signaling pathway in KYSE 170-STMN1 cells.

### Stathmin overexpression increases lung metastasis and xenografted tumor growth *in vivo*

To confirm the effect of stathmin overexpression on metastasis, we established a nude mouse model of metastasis *in vivo* to observe the lung metastasis ratio. STMN1 or control cells were injected into the tail vein of individual mice. After 39 days, three mice were euthanized. Two of the three STMN1-injected mice had developed metastatic nodules in the lung, whereas no metastasis was observed in the control group. After 49 days, all three mice in the STMN1 group had developed lung metastatic nodules, and two of the three mice injected with control cells had also developed metastatic nodules (Figure 6B). HE staining of the mouse lung tissue revealed that the size of the nodules in the STMN1 group was much larger than that of the control group (Figure 6C and 6D). Furthermore, mouse body weight was reduced in the KYSE170-STMN1 metastasis model relative to the control group (*P*<0.001) (Figure 6A). To investigate the tumor growth of stathmin-overexpressing cells *in vivo*, we evaluated tumor volume and weight in a mouse xenograft model. Mice bearing KYSE 170-STMN1 and control cells were inoculated for five weeks by subcutaneous injection, and no significant difference in body weight between the two groups were observed (Figure 7B). However, tumor volume and weight were greatly increased in the stathmin-overexpression group compared to those in the control group (Figure 7A and 7C). Tumors from STMN1 and control models were examined for markers of tumor cell proliferation (Ki67), keratin 17 and stathmin. As shown in Figure 7D, significant increases in stathmin, Ki67 and keratin17 in tumor tissues were observed in the STMN1 group. These data indicated that stathmin overexpression promoted ESCC cell growth and metastasis in the mouse *in vivo* model.

### Stathmin regulates ERK activation

**Figure 9:**
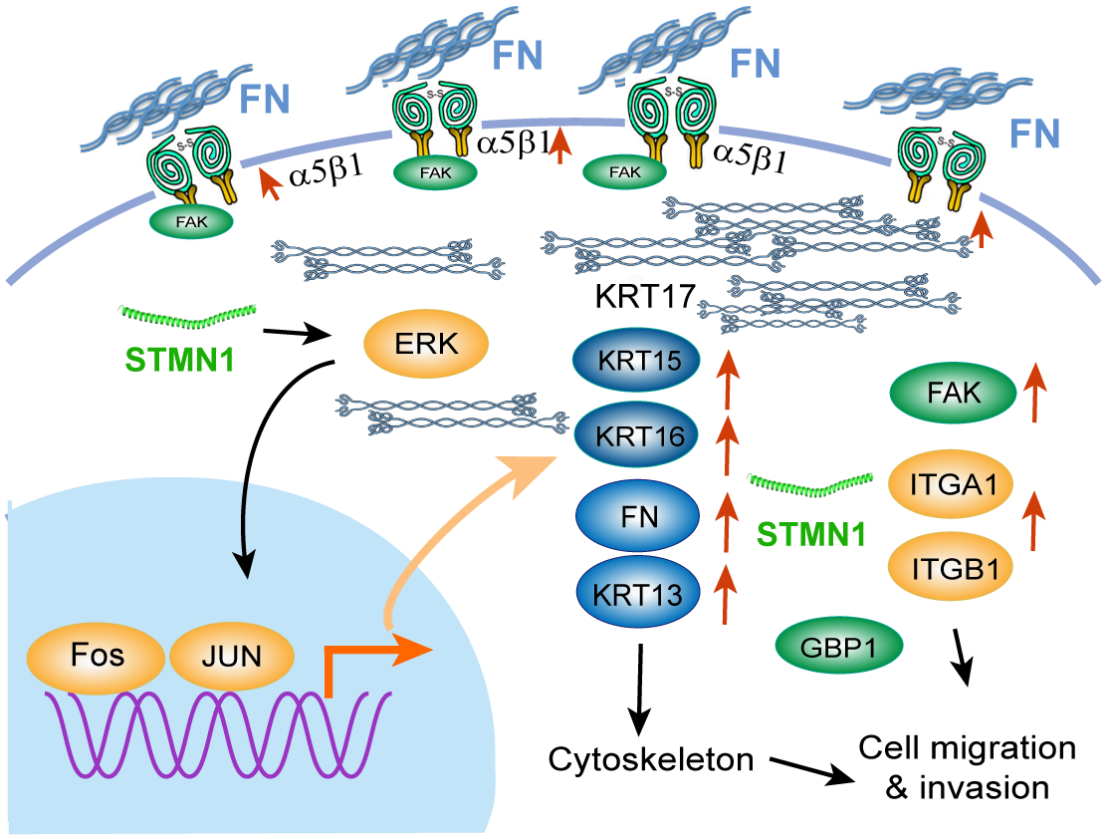
Illustration of the signaling pathway for ESCC cell migration induced by stathmin overexpression Overexpression of stathmin increased the number of cellular adhesion molecules and the level cytokeratin 17 of intermediate filaments, promoting cell invasion and migration via the FN/integrinα5β1/FAK signaling pathway. The red arrows indicate the upregulated genes.

As described above, stathmin overexpression promoted integrinα5β1/FAK expression and contributed to ESCC cell adhesion and migration *in vitro* and *in vivo*. To identify whether stathmin can affect the kinase activity of FAK downstream, we analyzed the phosphorylation levels of ERK, JNK, AKT, and mTOR by performing western blotting. The protein and phosphorylation levels of AKT, JNK and mTOR did not significantly differ between the STMN1 group and the control group (Supplementary Figure 5A-5C). In contrast, the P-ERK level was significantly increased in the STMN1 group compared to that in the control group. The total level of ERK did not differ between the two groups (Figure 8A). Real-time PCR analysis confirmed that the mRNA level of ERK downstream transcription factors, including FOS, EGR1 and JUN, were increased in the STMN1-group cells (Figure 8B). Knockdown of stathmin in KYSE 510 and KYSE 170-STMN1 cells resulted in a significant decrease of P-ERK levels (Figure 8E and 8F). To determine whether the ERK pathway activation was related to cell migration in the STMN1 group, we used the ERK phosphorylation inhibitor AZD8330 to treat the KYSE 170-STMN1 cells. The P-ERK levels were decreased after treatment with AZD8330 for 3 h. The *in vitro* wound-healing assay showed that the motility of the KYSE 170-STMN1 cells treated with AZD8330 was inhibited compared with that of the DMSO group (Figure 8C and 8D). Following knockdown of stathmin by siRNA, the protein levels of integrinα5β1, FAK and P-ERK were greatly reduced in the KYSE 510 and KYSE 170-STMN1 cells (Figure 8E and 8F). As illustrated in Figure 9, we propose a model to explain how stathmin overexpression promotes metastasis in ESCC cells: (i) the overexpression of stathmin increases the number cellular adhesion molecules and (ii) increases the keratin 17 of intermediate filaments for metastasis, (iii) promoting cell invasion and migration via the FN/integrinα5β1/FAK signaling pathway. The results indicate that stathmin overexpression influences ESCC cell invasion and migration via the integrinα5β1/FAK/ERK signaling pathway.

## DISCUSSION

Currently, the sensitivity and diagnostic value of serum markers for ESCC are low [6, 30–32]. In a previous study, our laboratory found that stathmin expression was significantly upregulated in ESCC, which might act as a biomarker for ESCC diagnosis and prognosis [21]. To investigate the levels of serum stathmin, we evaluated 535 ESCC patients and 288 healthy controls by ELISA. The results showed that the level of serum stathmin in ESCC patients was significantly higher than that in healthy controls (5.98±2.89 ng/ml vs. 2.16±1.19 ng/ml, *P* < 0.001), which indicated that high levels of stathmin may be particularly related to the malignant behavior of ESCC. We also observed that high levels of stathmin were positively correlated with lymph node metastasis, advanced TNM stage and tumor size in ESCC. These results suggested that stathmin has the potential to be a diagnostic marker for ESCC. Our findings are consistent with those of previous investigations reporting that overexpression of stathmin in ESCC tissue was associated with poor prognosis [19–22]. We found that stathmin acts as a marker for ESCC diagnosis with a sensitivity of 0.886, a specificity of 0.806, and a serum cutoff value of 3.014 ng/ml. Thus, overexpression of stathmin might promote oncogenesis and the development of ESCC, followed by cell motility, proliferation and metastasis of ESCC.

The *in vitro* experiments confirmed that overexpression of stathmin promotes ESCC cell proliferation, adhesion and metastasis; and the xenograft experiments demonstrated that stathmin overexpression increased mouse tumor burden and promoted lung metastasis of ESCC cells. Stathmin overexpression enhanced ESCC cell adhesion to the extracellular matrix by promoting the expression of integrinα5β1/FAK. In addition, integrinα5β1/FAK expression increased activation of the ERK pathway, promoted adhesion-related gene expression, and enhanced the migration of ESCC cells. These results support the notion that stathmin has a vital role in ESCC progression. Significantly elevated levels of stathmin have been detected in the urine and blood from urothelial carcinomas of the bladder using ELISA [33]. Mass spectrometry analysis of protein extracted from lung cancer cell culture medium revealed high levels of stathmin, which suggests that stathmin is released from lung cancer cells in some channels. Stathmin can be detected in ExoCarta, suggesting that it can be secreted from the cells [34]. Stathmin can be detected in the serum by ELISA, and serum stathmin protein levels were significantly elevated in ESCC patients and associated with lymph node metastasis and other clinical parameters. These results suggest that stathmin can be used as a serum biomarker for ESCC detection.

Cell movement contributes to cell membrane contraction, which depends on cell-extracellular matrix adhesion and dissociation. The cytoskeleton mainly consists of microfilaments, microtubules and intermediate fibers and regulates cell motility, cell cycle, intracellular signal transduction and other important biological processes. Stathmin plays an important role in the regulation of cytoskeleton dynamics. One stathmin protein can bind with two α, β-heterodimers to form a T_2_S complex and promotes α, β-heterodimer dissociation from the microtubule. Otherwise, stathmin binds to the free microtubule heterodimers, which inhibits their assembly to the microtubule tip [8, 9]. In neuroblastoma cells, changes in the level of stathmin expression regulate the level of activation of the RhoA/ROCK pathway and consequently modulate changes in microfilaments [35, 36]. In this study, we found that the mRNA levels of KRT17, KRT15 and KRT13 and the protein level of KRT17 in stathmin-overexpressing cells were significantly increased, suggesting that stathmin affects the levels of intermediate filament family members. These results indicate that stathmin affects not only the microtubule dynamics but also the entire cytoskeleton.

The *in vitro* experiments showed that stathmin promoted ESCC cell metastasis. To characterize the molecular pathways affected by stathmin in ESCC cells, we used the Human Transcriptome Array (HTA2.0) to perform microarray-based global gene expression profiling of cancer cells with exogenous forced stathmin levels. We found that the mRNA levels of adhesion-related molecules and FN were significantly increased in the STMN1 group, which suggest that stathmin may exert a strong impact on cell-extracellular matrix adhesion. To test this hypothesis, slides were coated with FN, which is the main component of the extracellular matrix, to evaluate cell-extracellular matrix adhesion ability. The results showed that adhesion capacity was significantly increased in the STMN1 group. Integrins, the main family of cell surface receptors, mediate cell-extracellular matrix adhesion and signal transduction [37–39]. Integrinα5β1 recognizes the extracellular matrix tripeptide (Arg-Gly-Asp, RGD) of FN. The extracellular receptors of integrinα5β1 are inhibited by specific inhibitors, which can reduce the angiogenesis and tumor cell metastasis of colon cancer [40–42]. FAK has two different functions: to serve as a scaffold protein to recruit other proteins and mediate their protein-protein interactions and to act as a protein kinase to phosphorylate downstream substrates. FAK, which acts as a scaffolding protein, plays an important role in promoting tumor growth and metastasis in anaplastic thyroid cancer [43]. The intracellular domain of integrins can recruit FAK and FAK coupled with the cytoskeleton to form stable focal adhesions [44, 45]. Treatment with lunasin (an Arg-Gly-Asp, (RGD) cancer suppressor polypeptide) inhibits the activation of the FAK/ERK/NF-κB pathway in integrinα5β1-overexpressing cells, inhibiting the invasion and metastasis of colon cancer. However, it is insensitive to cells with low integrinα5β1 expression [41, 46]. Enhance cell and extracellular matrix cross-talk, which can promote integrin clustering and elevate the expression of FAK, contributes to cell adhesion, proliferation and invasion [47]. In this study, we found that stathmin promotes invasion, metastasis and adhesion of ESCC cells, and we found that integrinα5β1/FAK protein levels were increased in STMN1 group. Silencing of FAK by specific siRNA significantly suppressed the adhesion, invasion and migration of KYSE 170-STMN1 cells. This finding suggests that extent to which stathmin promotes metastasis depends on the extent of integrinα5β1/FAK pathway activity increase in ESCC cells. In addition, the protein levels of integrinα5β1/FAK in stathmin-silenced cells were decreased, further demonstrating that the activation of the integrinα5β1/FAK pathway was affected by the level of stathmin protein.

The ERK pathway is a relatively mature pathway that regulates the expression of genes, cell proliferation, migration and other activities [48, 49]. The expression of FN is positively correlated with the invasion and metastasis of ESCC, which may be related to the activation of the ERK pathway [50, 51]. The levels of the ERK downstream transcription factors FOS, JUN, EGR1 were significantly increased in the STMN1 group, which indicated that the overexpression of stathmin increased activation of the ERK pathway. Integrin/FAK activates the Ras/ERK pathway, mediates cell-extracellular matrix adhesion and promotes cell proliferation [51–53].

In summary, this study demonstrated that STMN overexpression increased the number of cellular adhesion molecules and facilitated ESCC cell invasion and migration via the FN/integrinα5β1/FAK signaling pathway by increasing the amount of cytokeratin 17.

## MATERIALS AND METHODS

40, 6-diamidino-2-phenylindole (DAPI) was obtained from Sigma-Aldrich (St. Louis, MO).Stathmin, FAK, integrinα5/β1, Akt, phospho-Akt, ERK, phospho-ERK, phospho-S6, 4EBP1, phospho-4EBP1, FLAG, keratin17,JNK, and phospho-JNK antibodies were purchased from Cell Signaling Technology (Boston, MA). The anti-human CD49e integrinα5 blocking antibody was purchased from Biolegend (San Diego, CA). FAK inhibitor (PF-00562271), ERK inhibitor (AZD8330) and mitomycin C were purchased from Selleck Chemicals (Houston, TX). RPMI-1640 and Opti-MEM medium were purchased from Gibco-BRL (Grand Island, NY). Fetal bovine serum (FBS) was purchased from PAA (Pasching, Austria), and Lipofectamine 2000 was purchased from Invitrogen (Carslbad, CA).

### ELISA assay

Serum samples of pathology-confirmed esophageal squamous cell carcinoma were obtained from patients at the Cancer Hospital of Chinese Academy of Medical. In addition, serum from 120 patients with other cancers, comprising 30 hepatocellular carcinoma (HCC) patients, 30 colorectal cancer (CRC) patients, 30 gastric cancer (GC) patients and 30 head and neck cancer (HNC) patients were collected from the same hospital. Serum samples from healthy controls were collected from the Beijing Coal General Hospital of the health examination center. The ELISA kit was purchased from the US (Cloud-Clone company, No. SE-C892Hu). Serum samples and standards were diluted, added to the wells, and incubated at 37°C for 2 h. The liquid in the plate was removed, and 100 μl of working solution A was added to each well. The wells were then aspirated at 37°C for 1 h and washed 3 times. Then, 100 μl of reaction solution B was added to each well, followed by incubation at 37°C for 30 min. Then, the wells were washed 5 times, and 90 μl of substrate solution was added to each well, followed by incubation at 37°C for 20 min. Next, 50 μl of stop solution was added to each well. The absorbance at 450 nm was read from a blue-to-yellow plate reader (Bio-rad) according to the concentration and A-value of the kit standard. The stathmin concentrations in the serum samples were measured and analyzed statistically based on sample A 450. Each sample was repeated 3 times; the mean value was taken as the serum stathmin concentration of the sample.

### Cell lines and cell culture

The human ESCC cell lines KYSE 170, KYSE 30 and KYSE 510 were a generous gift from Dr. Y Shimada (Kyoto University, Japan). The cells were cultured in complete RPMI 1640 medium supplemented with 10% FBS, penicillin (100 U/ml), and streptomycin (100 mg/ml) at 37°C in a humidified incubator containing 5% CO_2_.

### Plasmid DNA transfection

Cell lines transfected with human STMN1 or empty vector pCMV6were established by transfection of pCMV6 vector (Origene) containing the full-length STMN1 cDNA or pCMV6 vector alone, respectively, into esophageal cancer cells KYSE 170 and KYSE 30 using Lipofectamine 2000 as per the manufacturer’s instructions. The stable clones were selected with 200 ug/ml G418.

### siRNA transfection

Prior to transfection, cells were plated in 6-well plates and allowed to reach 80% confluence. After 12–16 h of culture, 5 μl of Lipofectamine 2000 and 5 μl of duplexed siRNA (sequences:

si-FAK:5′-CCCAGGUUUACUGAACUUATT-3′, 5′-UAAGUUCAGUAAACCUGGGTT-3′;

si-STMN-1:5′-UCGCUUGUCUUCUAUUCACTT-3′, 5′-GUGAAUAGAAGACAAGCGATT-3′;

si-STMN-2:5′-CAAAGAAGAAGGAUCUUUCTT-3′, 5′-GAAAGAUCCUUCUUCUUUGTT-3′;

si-KRT17-2:5′-CCCACCUGACUCAGUACAATT-3′, 5′-UUGUACUGAGUCAGGUGGGTT-3′; and

si-KRT17-3:5′-GCGUACCAUUGUGGAAGAGTT-3′,

5′-CUCUUCCACAAUGGUACGCTT-3′;

GenePharma, Shanghai, China) were separately pre-incubated in 150 μl of Opti-MEM for 5 min. These two solutions were then mixed and incubated at RT 30 min. The cells were washed twice with 1×PBS and pre-incubated in 700 μl of Opti-MEM medium. The siRNA and liposome mixture was then added to the culture medium. After 6 h, the medium was replaced with culture medium containing 10% FBS. The cells were harvested after 48 h, and knockdown efficiency was verified by Western blot analysis.

### Western blot analysis

The total protein extracts from cells were prepared in ice-cold cell lysis buffer (50 mM Tris-HCl, pH 8.0, with 150 mM NaCl, 1.0% NP-40, 0.5% sodium deoxycholate, and 0.1% SDS) containing protease inhibitor (500 mM phenylmethylsulfonyl fluoride). The cell homogenate was spun at 12,000 rpm for 15 min at 4°C, and the protein concentration in the supernatant was determined by the Bradford assay. Approximately 30 μg of the supernatant was resolved by SDS-PAGE and transferred onto Millipore polyvinylidene difluoride membranes. The membranes were blocked for 3 h with 10% (w/v) nonfat dry milk in 1×PBS and incubated with primary antibodies diluted in 3% milk 1×PBS buffer at 4°C overnight. After washing with Tris-buffered saline (TBS) containing 0.1% Tween-20 (TBS-T), the blots were incubated for 1 h at RT with goat anti-rabbit/mouse IgG horseradish peroxidase-conjugated secondary antibody (1:3,000 dilution) in 3% milk 1×PBS buffer. The blots were again washed thrice with TBS-T for 15 min and then developed with ECL Western blotting detection reagent (GE Healthcare). All blots were reprobed for β-actin (1:3,000) or GAPDH (1:3,000) as an internal reference for protein loading. The band intensities of scanned blots were quantified using Image J. The integrated intensity of a fixed area was measured, and background levels were subtracted.

### Wound-healing assays

Cell monolayer were scraped with a sterile pipette tip and cultured in RPMI-1640 supplemented with 10% FBS. Wound closure was monitored for different times, and the cells were fixed and stained with Coomassie Brilliant Blue. The photographs represent one of three independent experiments (10× magnification, phase-contrast microscopy).

### Transwell migration assays

*In vitro* cells migration was measured in Transwell Chambers (5 μm; Corning Costar). Cells were seeded onto the upper chamber. Transmigrated cells were fixed and stained with crystal violet (AMRESCO). The cells were manually quantified by obtaining counts from five photos of random fields of the underlying membrane and calculating the mean number.

### Adhesion assay

Cell adhesion assay was performed on FN-coated 96-well plates or glass cover slips in 6-well plates, respectively. Briefly, cells were seeded with serum-free medium in the plates, and the cells were incubated at 37°C for different times to determine the adhesion to FN. The plates were then carefully washed twice with 1×PBS to remove non-adherent cells. Next, for the 96-well plates, fresh medium containing 10% FBS and 10% μl WST-8 was added to each well. The plates were incubated for another 3 h, and the relative numbers of attached cells were detected at 450 nm using an enzyme-linked immunosorbent assay (ELISA) reader. For the glass cover slips in 6-well plates, the attached cells were fixed and stained with crystal violet, and the irrelative numbers were manually quantified by obtaining counts from photographs and calculating the mean number.

### Colony formation assay

Cells were trypsinized and seeded in six-well plates (200, 500, or 1000 cells per well) and cultured in RPMI-1640 supplemented with 10% FBS. After 10 days, cells were fixed in 4% formaldehyde and stained with 0.25% crystal violet. Clones were washed and counted. All experiments were carried out in triplicate.

### Immunofluorescent staining

Cells growing in the logarithmic phase were seeded onto glass cover slips overnight, fixed with 4% (w/v) paraformaldehyde for 30 min, and rinsed with 1×PBS for 15 min. The cells were subsequently permeabilized with 0.1% (w/v) Triton X-100 at RT for 10 min, rinsed with 1×PBS, and incubated with 1×PBS containing 2% BSA for 30 min. Next, the cells were incubated for 1 h at RT with the appropriate primary antibodies, rinsed several times, and incubated at RT for 30 min with the corresponding fluorescent secondary antibodies. DAPI was used for nuclei detection. Fluorescence images were captured with a Nikon ECLIPSE 80i microscope.

### *In vivo* xenograft assay

Animal experiments were carried out as previously described [54]. For tumor engraftment, 6×10^5^ tumor cells were injected onto the right flanks or tail vein of female BALB/c nude mice (Harlan) in our pathogen-free animal facility. All experimental procedures using animals were previously reviewed and approved by the Institutional Animal Care and Use Committee (IACUC) at the Cancer Hospital of Chinese Academy of Medical Science. For right flank-injected nude mice, tumor volume was calculated using the formula 0.5×A× B^2^, where A is the length of the tumor, and B is the width.

### Immunohistochemical staining

For immunohistochemical staining, paraffin-embedded unstained slides from mice were incubated with an antibody against stathmin, Ki67, keratin17 or control immunoglobulin G (IgG; 1 mg/ml). After washing with 1×PBS, slides were incubated with a biotin-labeled secondary antibody. Signals were visualized using an ultrasensitive streptavidin–peroxidase system (Maxim Biotech).

### Microarray and real-time PCR analysis

Total RNA was isolated from untreated and stathmin overexpression KYSE 170 and parent cells using an RNeasy® mini kit (Qiagen). The quality of the total RNA was determined using a NanoDrop spectrophotometer (ND-2000; Thermo Fisher Scientific). RNA with an A260/A280 absorbance ratio ranging from 1.8 to 2.0 was used for cDNA synthesis. Gene expression profiles were analyzed on a GeneChip® Human Genome U133 Plus 2.0 array (Affymetrix, Santa Clara, CA), which contains 54,000 probe sets representing approximately 47,000 genes. The signal intensity of the gene expression was analyzed to generate CEL files using the default setting of Affymetrix® GeneChip® Command Console® 3.2 (AGCC) software. The Affymetrix Microarray Suite 5.0 (MAS5) and the Robust Multi-array Average (RMA) algorithm were used for the expression summary and signal calculation of the GeneChip® Human Genome U133 2.0 data [55], respectively. Differentially expressed genes were selected based on a >2.0-fold change and a q value <5%. Entrez gene identifiers were used to perform enrichment analysis using the Database for Annotation, Visualization and Integrated Discovery (DAVID) and the online database resource Search Tool for the Retrieval of Interacting Genes (STRING). Fold change was calculated relative to the average of the control group. The primer sequences are provided in Table 2 .

**Table 2:** Primers for qRT-PCR

GENE name	
KRT6C	Forward 5’ –GGATGCTTAGTGCCCTCACTT– 3’
	Reverse 5’ –GCTCAGCCTCAGAGAGAACAAT– 3’
KRT15	Forward 5’ –GGATGCTTAGTGCCCTCACTT– 3’
	Reverse 5’ –GCTCAGCCTCAGAGAGAACAAT– 3’
KRT17	Forward 5’ –GCTCAGCATGAAAGCATCCC– 3’
	Reverse 5’ –TTGTACTGAGTCAGGTGGGC– 3’
KRT13	Forward 5’ –GCTCAGCATGAAAGCATCCC– 3’
	Reverse 5’ –TTGTACTGAGTCAGGTGGGC– 3’
ARP2/3	Forward 5’ –GCGGCAAGGAAACATGACAG– 3’
	Reverse 5’ –CAGACGGGCTCTCAAATCCTT– 3’
GBP1	Forward 5’ –AGCCCTACAACTTCGGAACAG– 3’
	Reverse 5’ –TCTGGATTCGCCATCAGTCG– 3’
NT5E	Forward 5’ –GTATCCGGTCGCCCATTGAT– 3’
	Reverse 5’ –AAAGGCCTTCTTCAGGGTGG– 3’
GALNT5	Forward 5’ –AGCCCCGGAAGAGTCATAG– 3’
	Reverse 5’ –GTGCCCTCTCGTTTGCTACT– 3’
FN	Forward 5’ – ACAAGCATGTCTCTCTGCCAA– 3’
	Reverse 5’ –GCAATGTGCAGCCCTCATTT– 3’
LYPD3	Forward 5’ –CACGGACAATTCTCGCTGG– 3’
	Reverse 5’ –GCGGGTATGCACTCTCATTAC– 3’
CLCA2	Forward 5’ –AAAGTGACAGTGACCTCTCGC– 3’
	Reverse 5’ –GGCAGTGACAGTGGCATTAAG– 3’
FAK	Forward 5’ – AGCCGTTCGGGATTTTGCTA– 3’
	Reverse 5’ –TGTTGGGATGGTCGAACTGG– 3’
RASSF5	Forward 5’ –AGTAGCGCAGTCGCCAAA– 3’
	Reverse 5’ –GTCCAATAGTAGCGGGTACGG– 3’
CEACAM5	Forward 5’ –CTGTCCAATGACAACAGGACC– 3’
	Reverse 5’ –ACGGTAATAGGTGTATGAGGGG– 3’
ITGA5	Forward 5’ –TTACGGGACTCAACTGCACC– 3’
	Reverse 5’ –AGCCTGAAACACTCAGCCTC– 3’
ITGB1	Forward 5’ –CCTACTTCTGCACGATGTGATG– 3’
	Reverse 5’ –TCCCCTGATCTTAATCGCAAAAC– 3’
EGR1	Forward 5’ –TCGAGTTGGCAAAATGGGGT– 3’
	Reverse 5’ –TCACCATTGGTTTGCTTGGC– 3’
FOS	Forward 5’ –ATGGACCAGTGAAGCGATCAT– 3’
	Reverse 5’ –GTTCCTCCAAACTAGAAGCAGC– 3’
JUN	Forward 5’ –ACACGGCTTCGAGTCACTG– 3’
	Reverse 5’ –CGTCCTGGAAACCGTCCTTC– 3’

Total RNA was isolated from stathmin-overexpression KYSE170 and parent cells, and first-strand reverse transcription was performed using the SuperScript® III Reverse Transcriptase kit (Invitrogen, USA). Primers were designed with Primer-Blast software (http://www.ncbi.nlm.nih.gov/tools/primer-blast/). Amplification reactions were conducted using the SsoFastTMEvaGreen® Supermix with a CFX 96TM Real-Time System (Chemoscience, USA). GAPDH served as an internal control to normalize the loading of the template cDNA. Each experiment was repeated at least twice, and the fold change in gene expression was assessed using theΔCt method.

### Statistical analysis

Statistical significance was determined by Student's t test performed with or without Welch's correction for unequal variance using GraphPad Prism Version 6.05 (GraphPad Software, Inc., La Jolla, CA). Significance testing for tumor growth over time was performed using repeated measures two-way Anova with Tukey multiple comparisons of means as a post hoc test to evaluate differences between groups. In some cases, tumor volumes were compared at each time point using Student’s t test with the Holm-Šidák method to correct for multiple comparisons.

## SUPPLEMENTARY MATERIALS FIGURES


