# Supplementary material for: Overexpression of stathmin plays a pivotal role in the metastasis of esophageal squamous cell carcinoma
Source: Oncotarget. 2017 Jun 27;8(37):61742–60. doi: 10.18632/oncotarget.18687 (PMC5617461; doi:10.18632/oncotarget.18687)
Supplement: Supplementary file 1 [file oncotarget-08-61742-s001.pdf]

Overexpression of stathmin plays a pivotal role in the metastasis of esophageal squamous cell carcinoma

SUPPLEMENTARY MATERIALS

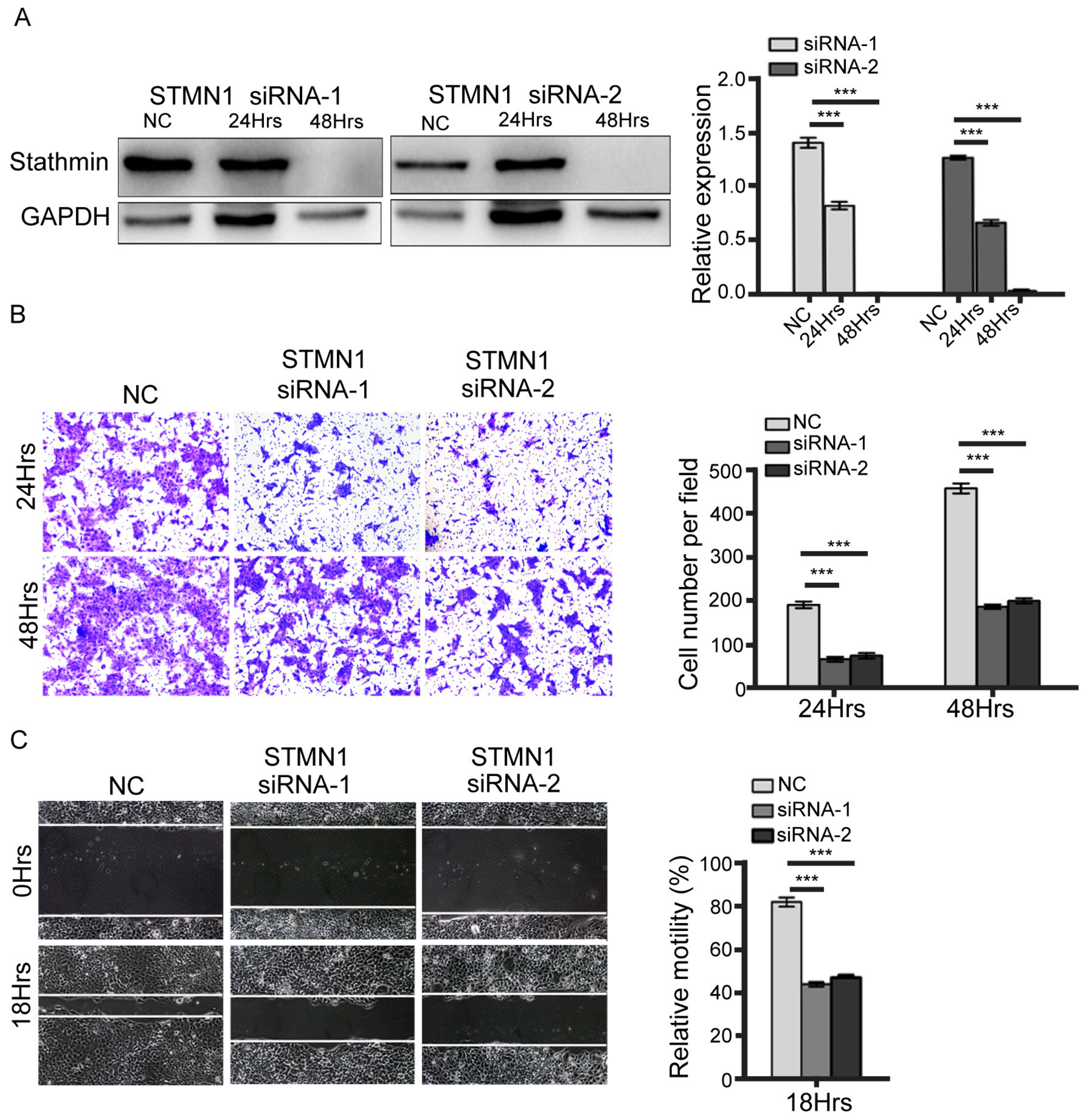

Supplementary Figure 1: Stathmin promoted ESCC cell invasion and migration. **(A)** Immunoblotting was used to analyze Stathmin levels in KYSE 510 cells. NC represents KYSE 510 cells transfected with negative siRNA; siRNA-1 and siRNA-2 represent KYSE 510 cells transfected with STMN1 specific siRNA-1 or siRNA-2, respectively. **(B)** The transwell invasion system demonstrated that compared with the NC, the silencing of Stathmin-expression inhibited invasion capacity of KYSE 510. The y-axis represents the number of invading cells. **(C)** Wound-healing assays were performed to investigate migratory potential of KYSE 510 after attenuated Stathmin levels. Quantitative migration assay results, in which the y-axis represents migration rates relative to NC cell, showed that Stathmin knockdown inhibited the migration of KYSE 510 cells. All assay were replicated, and the results are presented as the mean {plus minus} SD (***, P<0.001).

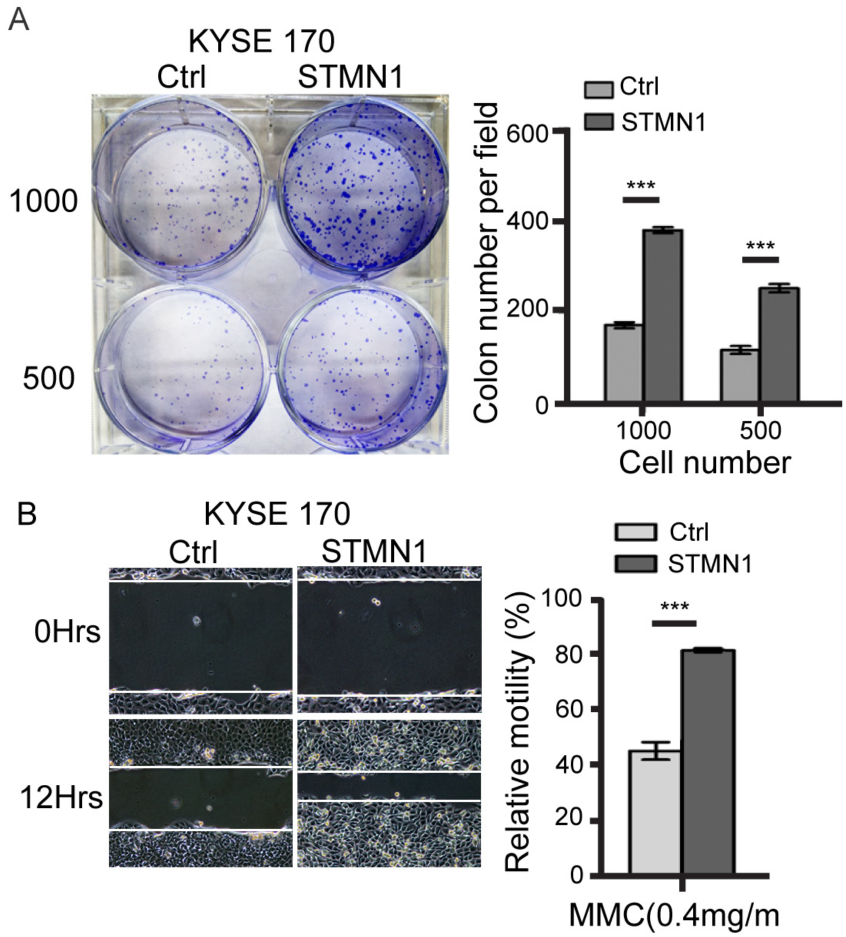

Supplementary Figure 2: Stathmin promoted ESCC cell proliferation. **(A)** Colony-formation assay was performed using six-well plates, the colony number of the STMN1 group was higher than that in Ctrl group. **(B)** The cells were seeded in six-well plates and cultured as a monolayer until they reached 100% confluency. Before scratch wounds,the cells were treated with MMC (0.4mg/ml) for 5min. Images of wounds were captured at different time points until the wounds closed completely (***, P<0.001).

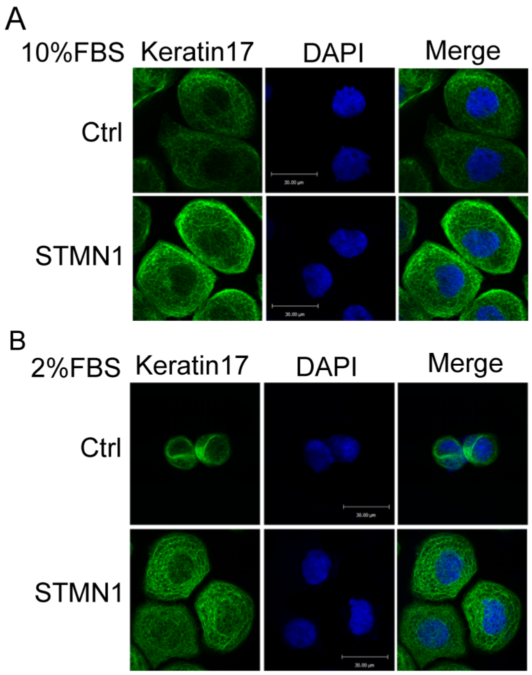

Supplementary Figure 3: The relationship between Stathmin and anti-starvation ability of ESCC cell. **(A)** In the normal medium culture condition, immunofluorescence staining of keratin17 in STMN1 group and Ctrl group. **(B)** After 2% FBS starvation treatment for 6 hours, immunofluorescence staining of keratin17 showed that the Ctrl cells showed keratin17 structural damage, while the STMN1 group remained intact.

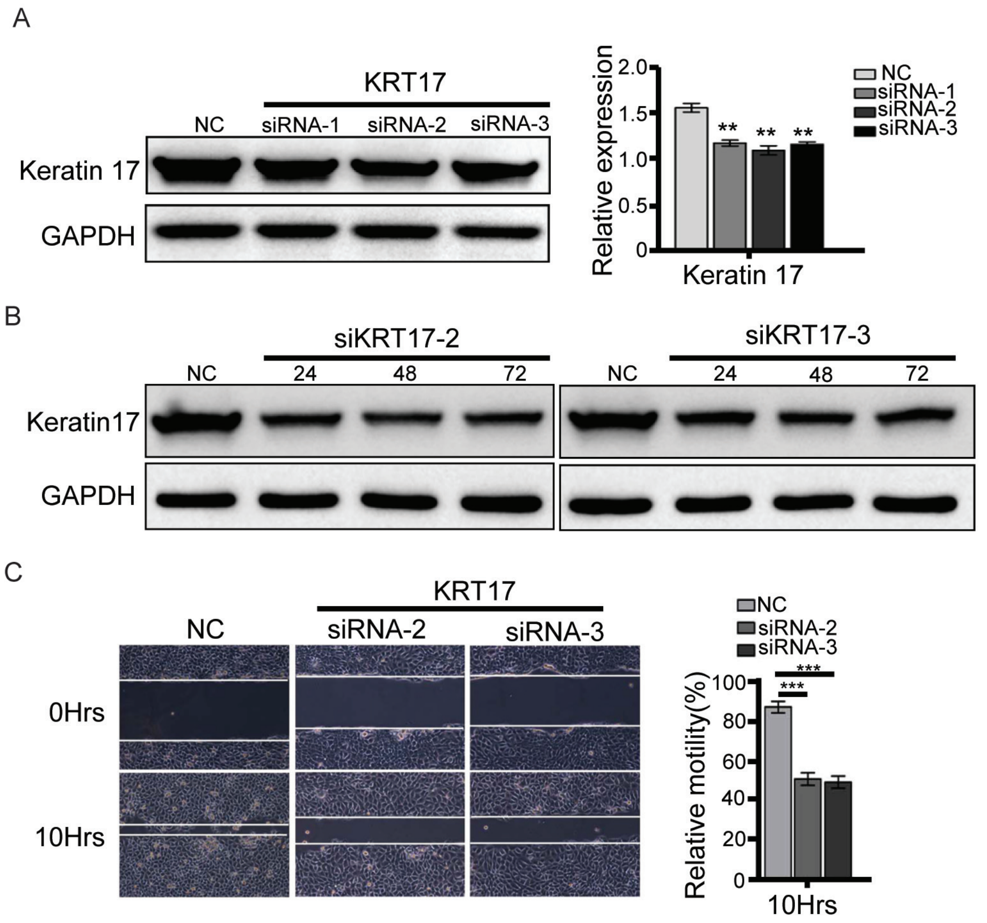

Supplementary Figure 4: Knockdown of KRT17 decreased cell mobility in KYSE 170-STMN1 cells. (A) Western blotting showed that KRT17-specific siRNA-1, siRNA-2 and siRNA-3 decreased Keratin17 expression. **(B)** Western blotting showed that KRT17-specific siRNA-2and siRNA-3 can knockdown Keratin17 expression after treated 72 hours. **(C)** The wound-healing assay was performed to detect the migratory potential of KRT17-knockdown KYSE 170-STMN1 cells and showed that the motility of the KRT17-knockdown group were decreased compared with that of the NC group.

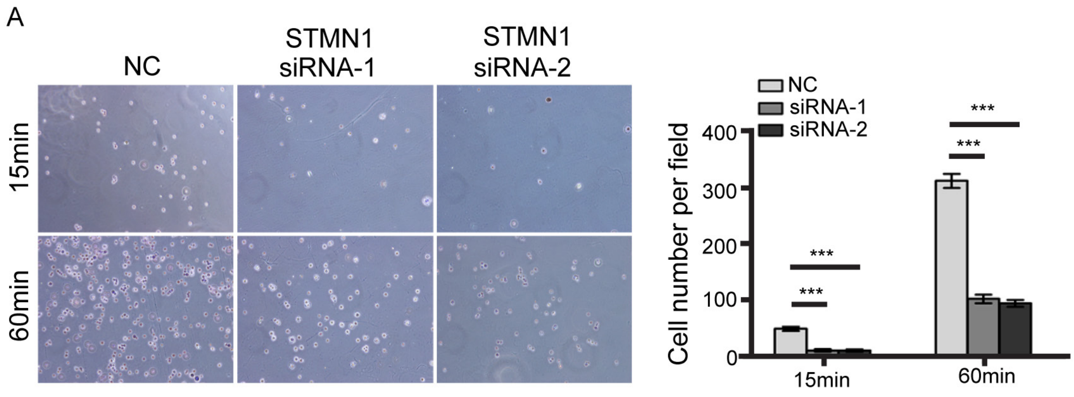

Supplementary Figure 5: Stathmin increases ESCC cell adhesion to FN. (A) Two different STMN1 specific siRNAs (shRNA-1 and siRNA-2) were used to knockdown the expression of Stathmin in KYSE 510 cells, and these cells adhesion ability evaluated by FN-coated slides. The results showed that the number of cells adhered to FN increased as time goes on; compared with the NC group, the siRNA group was significantly lower (***, P<0.001).

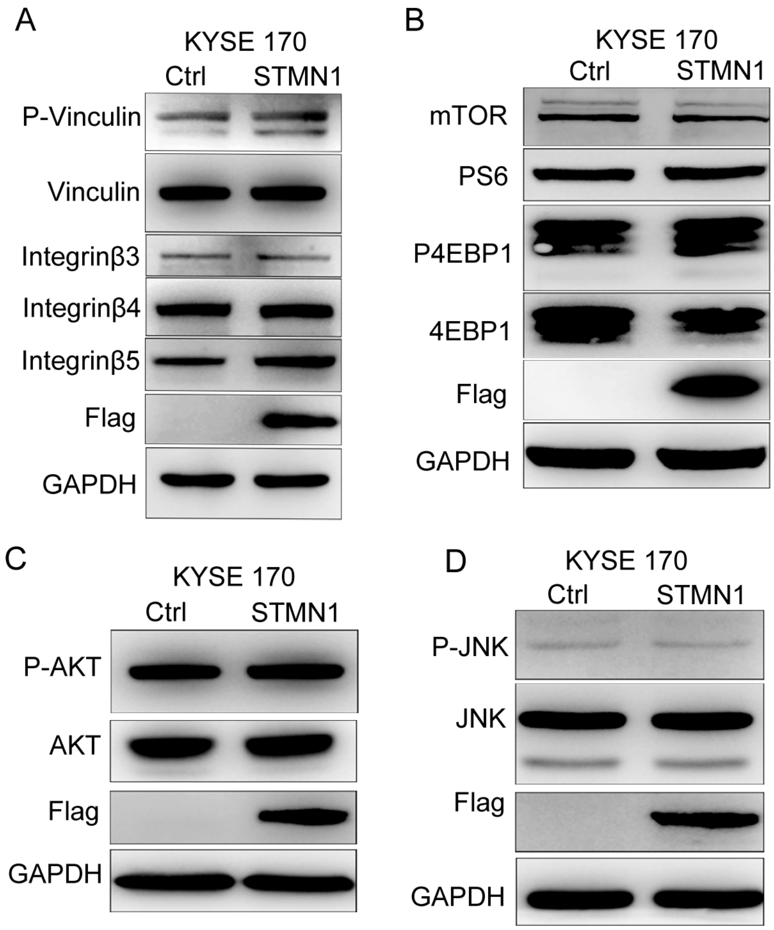

Supplementary Figure 6: Silencing of Stathmin affects intracellular signaling pathways. (A) The protein levels of integrin family other members such as integrinβ3, integrinβ4 and integrinβ5 and vinculin, P-vinculin and P-FAK were not changed in the two groups, GAPDH as the loading control. **(B)** Changes in stathmin expression did not affect the mTOR pathway activation level. **(C)** AKT and P-AKT protein levels were detected by immunoblotting, and there were no significant changes between the STMN1 group and the control group. **(D)** There were no significant changes in the levels of JNK and P-JNK after alter stathmin expression.
